# Current Research and New Perspectives of Telemedicine in Chronic Heart Failure: Narrative Review and Points of Interest for the Clinician

**DOI:** 10.3390/jcm7120544

**Published:** 2018-12-13

**Authors:** Emmanuel Andrès, Samy Talha, Abrar-Ahmad Zulfiqar, Mohamed Hajjam, Sylvie Ervé, Jawad Hajjam, Bernard Gény, Amir Hajjam El Hassani

**Affiliations:** 1 Service de Médecine Interne, Diabète et Maladies Métaboliques de la Clinique Médicale B, Hôpitaux Universitaires de Strasbourg, 1 porte de l’Hôpital, 67091 Strasbourg Cedex, France; 2 Equipe de recherche EA 3072 “Mitochondrie, Stress oxydant et Protection musculaire”, Faculté de Médecine de Strasbourg, Université de Strasbourg (Unistra), 4 rue Kirschleger, 67091 Strasbourg, France; samy.talha@chru-strasbourg.fr (S.T.); bernard.geny@chru-strasbourg.fr (B.G.); 3 Service de Physiologie et d’Explorations Fonctionnelles, Hôpitaux Universitaires de Strasbourg, 1 porte de l’Hôpital, 67091 Strasbourg CEDEX, France; 4 Service de Médecine Interne, Gériatrie et Thérapeutique, CHU de Rouen, 76000 Rouen, France; abzulfiqar@gmail.com; 5 Predimed Technology, 67000 Strasbourg, France; mha@newel.net; 6 Centre d’expertise des Technologies de l’Information et de la Communication pour l’autonomie (CENTICH) et Mutualité Française Anjou-Mayenne (MFAM), 49000 Angers, France; sylvie.erve@centich.fr (S.E.); jawad.hajjam@centich.fr (J.H.); 7 Equipe de recherche EA 4662 “Nanomédecine, Imagerie, Thérapeutiques”, Université de Technologie de Belfort-Montbéliard (UTBM), 25200 Belfort-Montbéliard, France; amir.hajjam-el-hassani@utbm.fr

**Keywords:** telemedicine, telemonitoring, artificial intelligence, information and communication technology, heart failure, chronic disease

## Abstract

Background: This is a narrative review of both the literature and Internet pertaining to telemedicine projects within the field of heart failure, with special attention placed on remote monitoring of second-generation projects and trials, particularly in France. Results: Since the beginning of the 2000’s, several telemedicine projects and trials focused on chronic heart failure have been developed. The first telemedicine projects (e.g., TEN-HMS, BEAT-HF, Tele-HF, and TIM-HF) primarily investigated telemonitoring or for the older ones, telephone follow-up. Numerous second-generation telemedicine projects have emerged in Europe over the last ten years or are still under development for computer science heart failure, especially in Europe, such as SCAD, OSICAT, E-care, PRADO-INCADO, and TIM-HF2. The E-care telemonitoring project fits within the telemedicine 2.0 framework, based on connected objects, new information and communication technologies (ICT) and Web 2.0 technologies. E-care is the first telemedicine project including artificial intelligence (AI). TIM-HF2 is the first positive prospective randomized study with regards to EBM with positive significant clinical benefit, in terms of unplanned cardiovascular hospital admissions and all-cause deaths. The potential contribution of second-generation telemedicine projects in terms of mortality, morbidity, and number of hospitalizations avoided is currently under study. Their impact in terms of health economics is likewise being investigated, taking into account that the economic and social benefits brought up by telemedicine solutions were previously validated by the original telemedicine projects.

## 1. Introduction

The rising prevalence of chronic diseases, e.g., chronic heart failure (CHF) or diabetes mellitus (DM), represents a real concern for public health [1]. For CHF, a prevalence of over 5.8 million affected people has been reported in the United States of America (USA), with over 26 million affected people worldwide. In France, more than one million people suffer from CHF, a large majority of those being elderly patients (1/3 ≥75 years old) [2]. Moreover, approximately 150,000 new heart failure (HF) cases are diagnosed every year. The cost of this chronic disease, which has recently skyrocketed, is estimated at several billion dollars in developed countries [1].

The management of these chronic diseases proves very challenging for healthcare professionals. They consume large amounts of medical resources at a time when a serious shortage in resources can be felt, along with medical deserts, where there is a lack of access to healthcare as well as other problems [1].

In this context, society must “reinvent the medicine of today and of tomorrow”.

To date, despite major therapeutic advances, most chronic diseases remain serious in terms of functional or survival prognosis, with high morbidity and mortality rates [3]. This applies particularly to CHF, with a five-year mortality rate based on the New York Heart Association (NYHA) at classes III-IV and is estimated to be between 20% and 40%, and approach that of metastatic breast cancer [2,3]. In France, the number of deaths directly related to CHF is estimated at 23,000 deaths per year [1].

Given this context, these patients frequently undergo emergency hospitalization and re-hospitalization with long hospital stays (with a mean duration of 9.6 days in France), resulting in an impaired quality of life [1,2]. In France, HF is responsible for more 210,000 hospitalizations per year [2], accounting for 5% of all hospitalizations, and is the main cause of hospitalization among elderly subjects.

Many of these hospitalizations could be avoided if patients took greater responsibility for their disease, were appropriately educated, and were better followed [3]. This last point has been particularly well-documented in the CHF context [4].

In this setting, telemedicine may be of significant help, given that it can be instrumental in optimizing CHF management, particularly by preventing emergencies such as acute HF and repeated iterative hospitalizations. Of note is that the re-hospitalization rate is 55% per year in France, which is the main evaluation end-point for most telemedicine studies [2,4]. Likewise, telemedicine may help make it possible to better structure integrated care pathways, again with the most important evidence to be found in CHF management [4,5] and, to a lesser extent, in that of other chronic diseases such as DM [6].

In this article, we have provided a narrative review of both medical and scientific literature as well as Internet research concerning telemedicine projects and studies, particularly those pertaining to second-generation telemedicine projects and studies developed in the CHF setting. We have restricted our searches to projects and studies using non-intrusive devices. Herein, we have reported on the experience gained with current telemonitoring projects in France.

## 2. First-Generation Telemedicine Projects and Studies

Since the beginning of the 2000’s, several telemedicine projects and trials have been conducted in the HF field [7,8,9,10,11,12,13,14,15,16,17,18,19,20,21,22,23,24,25,26,27]. Most of them have investigated “telemonitoring” or for the older ones “telephone follow-up”, as it is also known (Table 1), with a focus on CHF patients as defined under French legislation [28].

To the best of our knowledge, no completed projects have yet been published on “teleconsultation” and “teleexpertise” in the HF field, including an evaluation of the contribution of these two fields.

Several projects or studies have specifically investigated CHF subjects over 75 or over 80 years of age with good results in terms of ownership and system use [24,25].

It should be kept in mind that these projects and studies, particularly the earlier ones, more closely resembled a “telephone follow-up”, with care providers (e.g., nurses) traveling to patients’ homes, rather than the use of telemedicine as we think of it today with nonintrusive, automated, smart telemonitoring using remote sensors via modern communication technologies, Web 2.0 tools, or even artificial intelligence (AI) [4]. Therefore, in our opinion, these studies represent the “first generation” of telemedicine projects [4,22].

### 2.1. Original First-Generation Telemedicine Projects and Studies

As will be recalled, the results of telemedicine projects conducted so far in the CHF field differed from study to study, with fairly inconclusive results as far as potential clinical benefits in terms of re-hospitalization and decreased morbidity or mortality [7,8,9,10,11,12,13,14,15,16,17,18,19,20,21,22,23,24,25,26,27] and, in particular, the statistical significance of the results. As such, experts presently share widely divergent opinions on the actual utility of telemedicine in CHF patient management. 

It should be emphasized that the first studies on telemedicine for CHF sometimes conducted the following [7,8,9,10,11,12,13,14,15,16,17,18,19,20,21,22,23,24,25,26,27]:

- Inappropriate methodologies involving unsuitable patient groups, such as NYHA Class I and stable CHF patients without any representative control group, and having small sizes ranging from 50 to 1000 patients, and with short follow-up periods from three months to one year;
- Non well-structured organizations, with non-specialized staff for responding to alarms, and no association with patients’ general practitioners or cardiologists, nor any optimized management process or algorithm;
- Alarms being sounded too late and without therapeutic response;
- No educational programs;
- An absence of human interaction or contact.

Moreover, most studies were only based on weight fluctuations, did not include other warning or monitoring parameters, and had devices that were often under-utilized [7,8,9,10,11,12,13,14,15,16,17,18,19,20,21,22,23,24,25,26,27].

These points and limitations also explain most of the insignificant results of the first-generation telemedicine studies.

In our opinion, these limitations rendered the following: “any clinical benefits demonstrated illusory” [22].

### 2.2. Meta-Analysis of First-Generation Telemedicine Studies

Despite a number of limitations, several reviews and meta-analyses have demonstrated the undeniable utility of telemedicine [4,10].

To illustrate, in their review of “telephone follow-up” or “telemonitoring” of HF patients, Inglis et al. [10] revealed that telemedicine did have an impact on all-cause mortality, which fell significantly by 34% (*p* <0.0001) (A consensus was reached that a difference is “statistically significant” if the chance is less than 5 in 100 (*p* <0.05) for explaining the observed differences). In this study, the authors also reported that re-hospitalization for HF decreased by 20%, and that both patient quality of life and management costs improved with a well-accepted system. In this review, the total mortality, hospitalizations for all causes, and hospitalizations for HF were shown to have decreased respectively by: 13% (95% CI: 0.77–0.98), 5% (95% CI: 0.9–1) and 15% (95% CI: 0.77–0.93) in the “telephone support” studies (*n* = 9332), and 20% (95% CI: 0.68–0.94), 5% (95% CI: 0.89–1.01) and 29% (95% CI: 0.6–0.83) in the “telemonitoring” studies (*n* = 3860).

In the most cited meta-analysis, Anker et al. [4] reported that telemonitoring led to a reduction of all-cause mortality (10.4% vs. 15.4%; *p* <0.0001), all hospitalizations (47.2% vs. 52.1%; *p* = 0.02), and hospitalizations for HF (22.4% vs. 28.5%; *p* = 0.008).

### 2.3. Prospective Randomized First-Generation Studies with a Large Number of Patients

Therefore, several meta-analyses do suggest clinical benefits, whereas numerous prospectively clinical studies (e.g., Trans-European Network—Home-Care Management System (TEN-HMS), Better Effectiveness After Transition–Heart Failure (BEAT-HF), Telemonitoring to improve Heart Failure outcomes (Tele-HF), Telemedical Interventional Management in Heart Failure (TIM-HF), Interdisciplinary Network for Heart failure (INH), Weight monitoring In patients with Severe Heart failure (WISH) and The TElemonitoring in the Management of Heart Failure (TEMA-HF), including more than 3700 patients) have not individually confirmed this major advantage [4,8,9,10,13,22,23].

For example, four prospective randomized clinical studies, with a large number of CHF patients and a robust methodology (TEN-HMS, BEAT-HF, Tele-HF, and TIM-HF), reported individual results contradicting the previous results, and thus questioned the potential utility of telemedicine in CHF [8,9,12,23].

#### 2.3.1. TEN-HMS Study

In this setting, the Trans-European Network—Home-Care Management System (TEN-HMS) study, conducted in 2005, was the first large study that analyzed the role of telemonitoring in selected HF patients [8]. In this study, 426 patients (mean age of >70 years for more than 50% of the patients) were randomly assigned to telemonitoring, nurse telephone support, or standard care in a 2:2:1 ratio.

Telemonitoring allowed for data transfer (of weight, blood pressure, and ECG data) to a central *Web* server via a conventional telephone line, and then to workstations at each investigator site via a secure intranet connection. Patients were invited to transfer data twice a day. Values greater or lower than the predefined limits were automatically sent to study nurses who could either directly advise the patient or, in more severe cases, inform the primary care physician. In addition to standard care, patients belonging to the group with nurse telephone support were given the ability to contact the HF specialist nurse via telephone at any time during office hours. Additionally, the nurse contacted the patients via telephone every month in order to assess their symptoms and current medication, providing advice as necessary.

Home telemonitoring and nurse telephone support led to significantly lower mortality rates across the entire time period of the TEN-HMS study, compared to patients receiving care through traditional means. At 360-day follow-up, usual care patients on average lived 263 days, whereas home telemonitoring and nurse telephone support patients survived 303 and 307 days, respectively. In total, 27% fewer patients died after 240-day follow-up in the home telemonitoring group compared to those receiving usual care. Moreover, home telemonitoring led to an average 3.9-day reduction in hospital days per patient, measured at 240 days follow-up. For approximately 50% of patients hospitalized, there was a 6-day or 34% decrease in length of stay for patients in the home telemonitoring group. This resulted in a 10% overall cost savings for the home telemonitoring program relative to nurse telephone support. Patients in the home telemonitoring group reported positive feedback about their care. In total, 95% of these patients had a “very good or good” overall impression about their home telemonitoring experience.

#### 2.3.2. BEAT-HF Study

One of the first large studies focused on remote monitoring, the Better Effectiveness After Transition–Heart Failure (BEAT-HF) study, produced negative results after following 1437 patients (median age: 73 years) with cardiac decompensation [23].

In this study, no significant differences in all-cause readmissions within 180 days post-discharge (primary end point) were observed between the intervention group (which included pre-discharge HF education, regularly scheduled telephone coaching and remote monitoring of weight, blood pressure, heart rate and symptoms) and the standard care group. All-cause readmissions within 180 days post-discharge occurred in 50.8% (363 of 715) patients from the intervention group vs. 49.2% (355 of 722) of those from the control group (adjusted hazard ratio: 1.03 (95% CI: 0.88–1.20); *p* = 0.74).

In secondary analyses, 30-day readmission or 180-day mortality did not significantly differ between the groups. A significant difference in 180-day quality of life was observed between the intervention and standard care groups, with the better quality of life observed in the intervention group. There was no significant difference detected in unadjusted (*p* = 0.56) or adjusted (*p* = 0.63) analyses between the proportion of intervention participants (22.7% (162 of 715)) and usual care participants (21.6% (156 of 722)) with 30-day all-cause readmission. There was no significant difference detected in unadjusted (*p* = 0.34) or adjusted (*p* = 0.30) analyses between the proportion of intervention participants (14.0% (100 of 715)) and usual care participants (15.8% (114 of 722)) with 180-day all-cause mortality. There was a significant difference in 180-day quality-of-life scores between the intervention participants (mean: 28.50) and the control participants (mean: 32.63) in unadjusted (*p* = 0.02) and adjusted (*p* = 0.02) analyses. No adverse events were reported.

Adherence to the BEAT-HF intervention appears to have been a critical factor. Only 61.4% (439 of 715) and 55.4% (396 of 715) of patients randomized to the intervention were more than 50% adherent to telephone calls and telemonitoring within the first 30 days.

Based on the authors and in our opinion, this “average” adhesion of patients probably explains the results observed in the BEAT-HF study.

#### 2.3.3. Tele-HF Study

In the Telemonitoring to improve Heart Failure outcomes (Tele-HF) study, 1653 patients (median age: 61 years) hospitalized for CHF were randomized to either telemonitoring with voice-based interactive structured telephone support (*n* = 826) or standard care (*n* = 827) [9]. Of these patients, 70.6% had a depressed left ventricular ejection fraction (LVEF, <40%). Diabetes mellitus, hypertension, and coronary artery disease were the most common coexisting conditions, and 46% of patients had chronic kidney disease. Patients in the intervention group were invited to call a toll-free telephone number and answer a series of questions regarding their general health, weight and HF symptoms on a daily basis. A clinician then analyzed this information.

**Figure 1:**
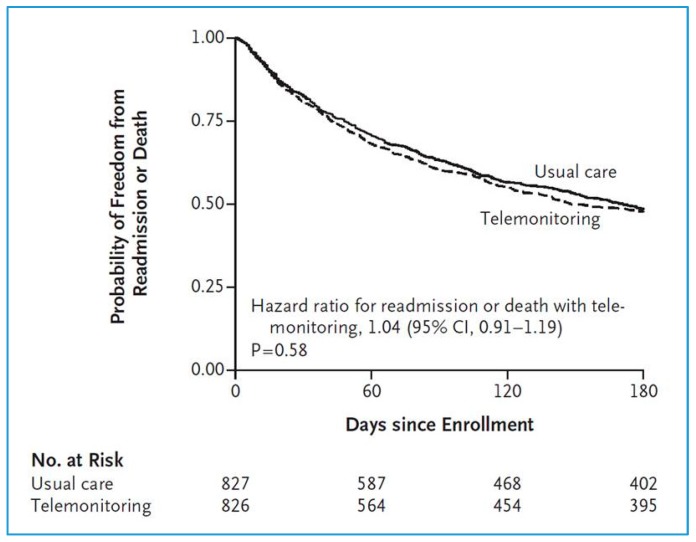
Tele-HF study (*n* = 1653). Probabilities of readmission for any reason or death for any cause (adapted from [9]).

No significant differences were revealed between the telemonitoring and standard care groups in terms of all-cause readmissions or all-cause mortality in the 180 days post-inclusion (primary end point) (Odds Ratio (OR): 1.04 (95% CI: 0.91–1.19)) [9]. The primary observation was that all-cause readmission or death within 180 days post-enrollment occurred in 52.3% (432 of 826) of the telemonitoring group patients vs. 51.5% of the standard care group (436 of 827) (Figure 1).

Readmission due to any cause occurred in 407 patients (49.3%) in the telemonitoring group and 392 patients (47.4%) in the usual-care group (difference, 1.9 percentage points; 95% CI, −3.0 to 6.7; *p* = 0.45). A total of 92 patients (11.1%) in the telemonitoring group and 94 patients (11.4%) in the usual-care group died during the 180-day study period (difference, −0.2 percentage points; 95% CI, −3.3 to 2.8; *p* = 0.88). Readmissions for HF, the number of days in the hospital, and the number of readmissions were also similar in the two groups.

However, adherence proved to be poor in spite of system-generated reminders. In fact, 14% of patients in the study’s telemonitoring group never used the system. Among the 85.6% of patients that made at least one call, adherence to the intervention was highest, 90.2%, during the first week of the study period and decreased to 55.1% by week 26. By the final week, only 55% of patients were actually using the system at least three times a week.

#### 2.3.4. TIM-HF Study

The TIM-HF study conducted in Germany involved 710 stable CHF patients (mean age: 66.9 years) randomly assigned to one of the following two groups: 1) telemonitoring by means of remote monitoring and telephone support (*n* = 354) and standard care (*n* = 356) [12]. The included patients had Class II or III NYHA stable CHF and a left ventricular ejection fraction of ≤35%.

Patients were provided with a personal digital assistant (PDA) with a wireless Bluetooth interface. The system collected ECG data, blood pressure readings and body weight, which were then communicated wirelessly to a central location with a physician available 24 h a day, 7 days a week.

**Figure 2:**
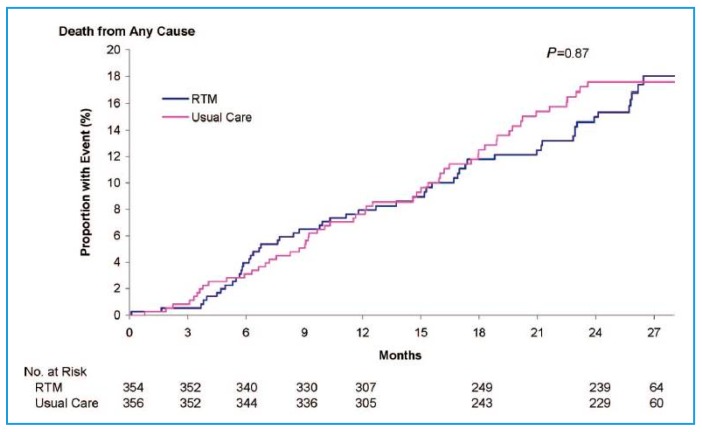
TIM-HF study (*n* = 710). Probability of death due to any cause; RTM = Remove Telemonitoring (adapted from [12]).

In this study, the all-cause mortality rate (primary end point) was 8.4 per 100 patient years of follow-up in the telemedicine group and 8.7 per 100 patient years of follow-up in the standard care group, without significant difference (OR: 0.97 (95% CI: 0.67–1.41); *p* = 0.87) (Figure 2) [12]. The TIM-HF study proved, however, to be insufficient in detecting any significant difference in mortality between groups.

For the composite secondary outcome, cardiovascular death and hospitalization for HF, the rate per 100 person-years of follow-up was 14.7% in the telemonitoring group compared with 16.5% in the usual care group (hazard ratio in the telemonitoring group, 0.89; 95% CI, 0.67 to 1.19; *p* = 0.44). This secondary outcome highlighted the stable nature of HF patients recruited into the study.

The likelihood of being in a better NYHA functional Class and having an improved PHQ-9 depression score at months 12 and 24 was similar between the assigned groups (*p* >0.5). Patients randomly allocated to the telemonitoring group compared with the usual care group showed an improved score for SF-36 physical functioning over the entire study period (*p* <0.05). This mean score at month 12 was 54.3 ± 1.2 vs. 49.9 ± 1.2 (*p* = 0.01); mean score at month 24 was 53.8 ± 1.4 vs. 51.7 ± 1.4 (*p* = 0.30).

The results of the TIM-HF study suggest that telemonitoring compared with usual care does not improve survival in stable, optimally treated patients with CHF.

### 2.4. 2016 ESC Guidelines

The 2016 European Society of Cardiology (ESC) guidelines for the diagnosis and treatment of acute and chronic HF were the first to recommend remote patient monitoring of CHF patients with a recommendation of Grade IIb and level of evidence B [27].

In this setting, telemonitoring is mainly focused on predicting acute decompensation episodes, usually associated with fluid congestion, which require therapy optimization such as an up-titration of angiotensin-converting enzyme inhibitors and beta-blockers. Clinical practice guidelines on CHF recommend daily weight recording, with weight increases of >2 kg per day defined as a warning alert [27].

### 2.5. Focus on Economic Considerations

In addition to these medical considerations, it is worth noting that all studies seem to agree that using telemedicine solutions in CHF management proved at least economically beneficial [7,8,9,10,11,12,13,14,15,16,17,18,19,20,21,22,23,24,25,26,27]. Depending on the study, the savings were estimated at between $5000 and >$50,000 per year per patient, according to the CHF stage and study setting.

In the study by Scalvini et al. [7], costs of CHF patient management fell by 24%, and hospital costs by €45,186 per year per patient.

In this context, the study by Burdese et al. [25] most convincingly illustrates the utility of telemonitoring in elderly CHF patients. In this study, a significant reduction was observed in re-hospitalizations (35 without vs. 12 with telemedicine, *p* = 0.0001), emergency department visits for an acute HF episode (21 vs. 5/year, *p* = 0.0001) and management costs (€11,656 vs. €40,065/year). Interestingly, only 8.6% of patients discontinued telemonitoring, thereby demonstrating that it was well accepted.

### 2.6. Focus on the Geriatric Population

For the most part, the patients included in the studies that we have just reviewed were not representative of the geriatric population [7,8,9,10,11,12,13,14,15,16,17,18,19,20,21,22,23,24,25,26,27]. An insignificant number of them were over 75 years old. In addition, most patients were elderly subjects in very good health with little comorbidity.

Yet, some studies published in the literature have included patients over 80 years old [24,25,26]. However, these studies yielded no conclusive results in terms of morbidity, mortality, and re-hospitalization for other causes. That said, the results confirmed that telemedicine was feasible in the geriatric population, and that the telemedicine solutions offered were employed.

The study by Burdese et al. [25] clearly illustrated the utility of telemonitoring in elderly HF patients. These authors confirmed its usefulness in 48 subjects with a mean age of 80.4 ± 7.7 years, suffering from severe and refractory HF and followed over the course of 20 months. There was a significant decrease in re-hospitalization (35 without vs. 12 with telemedicine, *p* = 0.0001), in visits to the emergency department for an acute episode of HF (21 vs. 5/year, *p* = 0.0001) and in management of costs (€116,856 vs. €40,065/year). Interestingly, only 8.6% of patients discontinued telemanagement, thus providing evidence that telemedicine was well accepted.

## 3. Second-Generation Telemedicine Projects and Studies

Over the last ten years, second-generation telemedicine projects and studies have emerged in the HF field, particularly in Europe [29,30,31,32,33,34,35]. These second-generation projects and studies address the shortcomings of those previously carried out (see above).

Several of these projects are include in the field of “telemedicine 2.0”, given that they all utilize new information and communication technologies (ICT) as well as Web 2.0 (tools for “e-Health 2.0”) [36].

In Europe, especially in France, Italy, Spain and Germany, numerous experiments on CHF telemanagement have been conducted or are still ongoing [35].

### 3.1. Focus on the Tools of Second Generation Telemedicine Projects and Studies

Most second-generation projects and studies rely on the standard connected tools for monitoring HF, such as blood pressure meters, heart rate monitors, weighing scales and pulse oximeters, which relay the collected information via Bluetooth, 3G or 4G [22,36,37].

Most of these projects also incorporate the following:

- Self-administered medical questionnaires or forms (symptoms and signs of HF);
- Tools for medical education, particularly disease self-appropriation, food hygiene, and physical activity (e.g., serious game);
- Tools for patient motivation (e.g., serious game);
- Tools for therapeutic (e.g., electronic pillbox) and hygiene observance;
- Tools for interaction between the patient and healthcare professionals like telephone support centers, tablets, and websites [36,37].

Thus, several projects provide questionnaires or forms regarding HF signs and symptoms (Table 2) [27] such as dyspnea, palpitations, edema, and fatigue corresponding to the acronym EPOF, namely, “Essoufflement, prise de Poids, oEdèmes, and Fatigue”, as experienced by the patient [38].

Several projects also include ECG monitoring and even video-conferencing [36,37].

A few of these telemedicine projects use machine learning in order to predict patient risks of acute HF or acute CHF decompensation [39,40]. These projects also include ICT and Web 2.0 technologies. In this setting, the cloud-based software aggregates, cleans and analyzes patient data to identify patterns that may indicate potential risks and to provide predictive insights on healthcare outcomes, such as the software MyPredi^TM^ (version 2) for the E-care project (see below) [37,41].

For these projects, several tools have been developed and used, such as artificial neural network (ANN) algorithms, data mining software and ontology [40]. In this context, three clinical datasets are of particular interest: (1) patients’ phenotypes, (2) patients’ electronic medical records containing physicians’ notes, laboratory test results, as well as other information on diseases, treatments, and epidemiology that may be of interest for association studies and predictive modeling on prognosis and drug responses, and (3) knowledge from literature, including rules on HF management [41].

In addition to these tools, it must be emphasized that HF telemonitoring may use implantable invasive devices that send data either intermittently or continuously to the receiving physician (e.g., automatic telemonitoring). These are outside the scope of this paper [42]. In CHF, implantable telemonitoring devices for multiple parameters or cardiac hemodynamic activity monitoring have recently proven to be an effective means for preventing frequent hospitalizations.

### 3.2. Original Second-Generation Telemedicine Projects and Studies in France

The main second-generation telemedicine projects presently undergoing development in France are:

- The SCAD project: “Suivi Cardiologique à Distance” (remote cardiac follow-up), first initiated in 2005, deployed in low Normandie between 2009 April and May 2012 and developed by the Caen University Hospital [29];
- The PIMPS project: “Plateforme Interactive Médecins patients Santé” (doctor-patient interactive health platform), initiated in 2013 and developed by the René-Dubos Hospital in Pontoise [30];
- The OSICAT project: “Optimisation de la Surveillance ambulatoire des Insuffisants Cardiaques par Télécardiologie” (optimization of outpatient monitoring in HF patients using telecardiology), initiated in 2012 and involving twelve local investigation centers coordinated by the Toulouse University Hospital [31];
- The MEDICA project: “Monitorage Electronique à Domicile de l’Insuffisance Cardiaque chronique” (electronic home-monitoring of CHF), initiated in 2014, developed by the REUNICA domicile and GMC-solutions santé groups working on the social protection of the elderly [32];
- The E-care project: “Détection des situations à risque de decompensation cardiaque chez les patients insuffisants cardiaques de stade III de la NYHA” (detection of risk situations for cardiac decompensation in HF patients with NYHA Class-III disease), initiated in 2014, whose medical aspects were developed by the Strasbourg University Hospital [22,33];
  The PRADO-INCADO project: A project which is scheduled to study the E-care platform for at-home monitoring of CHF patients (being deployed in the Strasbourg region) [43]. It is run by a group that assembles professionals from the Strasbourg University Hospital (Hôpitaux Universitaires de Strasbourg), the East Regional Health Agency (Agence Régionale de Santé du Grand Est), the Bas-Rhin branch of France’s National Health Insurance (Caisse Primaire d’Assurance du Bas-Rhin) and the Predimed Technology start-up. This project likely allows for an in-depth study for improving diagnoses via machine learning and detecting abnormalities in CHF patients at an early stage.

These projects have centered on either cohorts of well-defined and well-documented HF patients or prospective studies [22,29,30,31,32,33,43]. They have assembled relatively large patient samples, with most of them fulfilling the criteria of evidence-based medicine (EBM) with clinical relevant end points as primary and secondary criteria (e.g., HF hospitalization, HF mortality, medical costs, etc.).

All these projects are conducted using the second-generation telemedicine tools discussed above. The PIMPS project additionally includes laboratory monitoring of natriuretic peptides [30].

To our knowledge, the E-care project and the PRADO-P INCADO project are the only projects to include ICT, Web 2.0 technologies and AI tools, particularly the MyPredi^TM^ software (telemedicine 2.0) [33,43]. These latter projects have been developed in an effort to optimize home-monitoring of CHF patients. Regarding the patient phenotype, the system detects instances where there is a risk of cardiac decompensation and re-hospitalization (“personalized medicine”). In this context, MyPredi^TM^ automatically generates indicators of a worsening of the patient’s HF status (“predictive medicine”) [33,43]. In this context, it must be underscored that the E-care telemonitoring solution (with MyPredi^TM^ software) is currently being assessed for the “EC medical device” mark (proving its excellent quality), rather than just the simple “EC” mark, as is the case with numerous devices currently on the market.

All of these projects or studies satisfy the conditions of telemedicine, as laid out in Article 36 de la Loi de Financement de la Sécurité Sociale (Article 36 of the Social Security Financing Act) [28,29,30,31,32,33,43].

### 3.3. Focus on the Results of Original Second-Generation Telemedicine Projects and Studies Conducted in France

#### 3.3.1. SCAD Project

In the SCAD (“Suivi Cardiologique à Distance”) project, 90 patients were randomly selected from 2009 April to 2011 May (*n* = 45 for each group) (extracted data from the Thesis of the Faculty of Medicine from Caen, France and [29]). The population was elderly, with a mean age of 78 ± 6 years, mostly male (78%) and at high risk of re-hospitalization (mean brain natriuretic peptide (BNP) level of 1025 ± 950 pg/mL).

At twelve months, 1040 days of hospitalization for acute HF were recorded. Monitoring by educational telemedicine significantly reduced the number of hospital days for acute HF: 590 days in the control group vs. 450 days in the telemedicine group (*p* = 0.044).

The criterion “death or hospitalization for acute HF” occurred less frequently in the telemedicine group, specifically 57.8% in the control group vs. 35.6% in the telemedicine group (*p* <0.05). During HF re-admissions, patients receiving telemedication exhibited lower intra-hospital mortality: 18.2% vs. 0% (*p* < 0.02).

#### 3.3.2. E-Care Project

The E-care (“Détection des situations à risque de decompensation cardiaque chez les patients insuffisants cardiaques de stade III de la NYHA”) telemonitoring project, conducted in Strasbourg [22,33,44], falls under the “telemedicine 2.0” category [36]. It was developed and designed to optimize home monitoring of CHF patients by detecting, via a telemonitoring 2.0 platform, situations having a risk of cardiac decompensation and re-hospitalization. The AI of the E-care platform (MyPredi^TM^) automatically generates indicators of “health status” deterioration, i.e., “warning alerts” for any chronic disease worsening, particularly CHF, that may lead to hospitalization if not treated properly. To our knowledge, this is the first project to use AI in addition to ICT.

**Figure 3:**
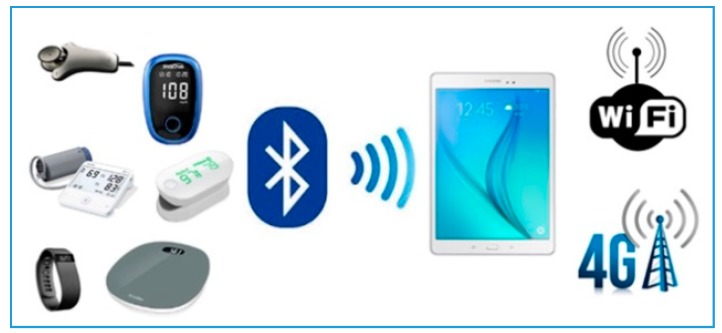
E-care’s connected non-intrusive medical sensors.

The platform consists of connected, non-intrusive medical sensors (Figure 3), a touchscreen tablet connected to Wi–Fi, and a router or 3G/4G, making it possible to interact with the patient and provide education on treatment, diet and lifestyle [33].

**Figure 4:**
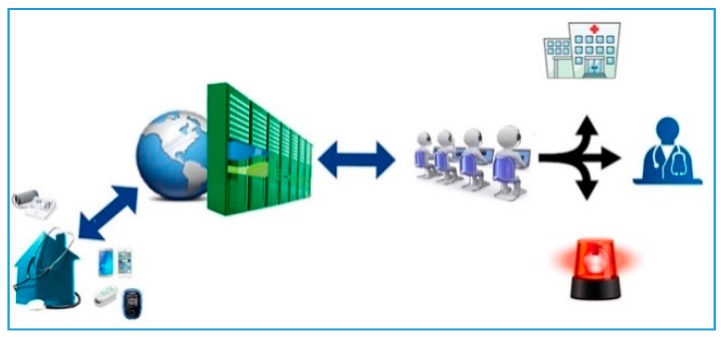
E-care platform.

**Figure 5:**
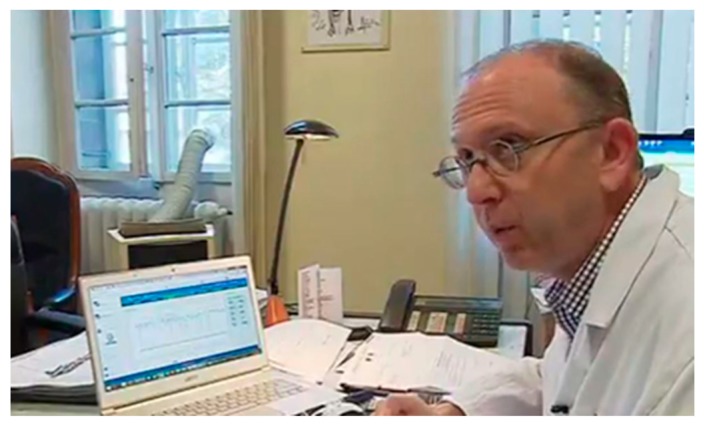
Health professional consults the E-care’s Internet portal.

The system (Figure 4) involves a server that hosts the patient’s data and a secure internet portal to which the patient and hospital as well as non–hospital based healthcare professionals can connect (Figure 5) [7].

E-care is based on a smart system consisting of an inference engine and medical ontology for personalized synchronous or asynchronous analysis of data that is specific to each patient with, as necessary, the sending of an AI-generated alert (MyPredi^TM^).

Between February 2014 and April 2015, 175 patients were included in the E-care project [33]. During this period, the E-care platform was used on a daily basis by patients and healthcare professionals, according to a defined protocol of use specific to each patient. The mean age of these patients was 72 years, and the ratio of men to women was 0.7. The patients suffered from multiple concomitant diseases, with a mean *Charlson index* (comorbidity index validated and often used for the elderly) of 4.1. The five main diseases were: CHF (in more than 60% of subjects), anemia (in more than 40% of subjects), atrial fibrillation (in 30% of subjects), type 2 diabetes (in 30% of subjects) and chronic obstructive pulmonary disease (COPD, in 30% of subjects).

During the study, 1500 measurements were taken for these 175 patients, which resulted in the E-care system generating 700 alerts for 68 patients [44]. Some 107 subjects (61.1%) had no alerts during follow-up. Follow-up data analysis of these 107 patients revealed that they exhibited no clinically significant events that might eventually have led to hospitalization. Analysis of the warning alerts showed that the E-care platform automatically and non-intrusively detected a worsening of the patient’s health, particularly HF decompensation (between two to nine days), with a sensitivity, specificity, as well as positive and negative predictive values of 100%, 72%, 90% and 100%, respectively. Both the healthcare professionals and patients, even the frailest, used the E-care system without difficulty until the end of the study. For non-autonomous patients, the system was employed by a nurse in addition to his or her other assigned tasks, such as bathing and administering medication, or by family members and others close to the patient.

Of particular note is that the tools and the system were tested and improved beforehand by both patients and the personnel at the Centre National des Technologies de l’Information et de la Communication pour le Handicap (CENTICH, Angers), namely, the French research center, in using ICT for promoting autonomy in the elderly. Hence, based on our experience, age is not a limiting factor for using new technologies. Several recent studies have reached similar conclusions through documenting the use of telemedicine solutions, even among 80-year-olds [24,25,42].

#### 3.3.3. OSICAT Project

The OSICAT (“Optimisation de la Surveillance ambulatoire des Insuffisants Cardiaques par Télécardiologie”) project, launched in 2013, consists of 870 patients divided into two groups: a group receiving remote home monitoring (*n* = 435) and a control group receiving standard care (*n* = 435) [31]. The results, which are expected in 2018–2019, will also assess medical efficacy and cost-effectiveness.

The mean age of the patients included was 70 years (with 26.7% of patients being ≥80 years old [31]), and the ratio of men to women was 2.5. Overall, 81.6% of patients were classified as NYHA Classes II-III (10.5% were Class IV). CHF was related to systolic dysfunction (LVEF <45) in 63.6% of patients. Only 24.9% of the OSICAT patients have benefited from education therapy, as mentioned in the protocol.

#### 3.3.4. PRADO-INCADO Project

As discussed above, the E-care platform, with MyPredi^TM^ AI software, appears capable of preventing hospitalization through early detection of CHF deterioration and the subsequent warning of the patient’s caregivers, thus allowing them to intervene [43].

**Figure 6:**
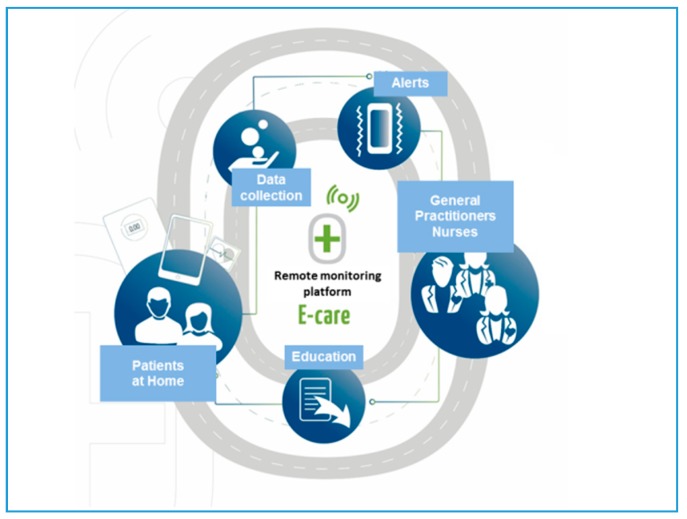
The PRADO INCADO project. This project employs the E-care smart telemonitoring platform and MyPredi^TM^ artificial intelligence to follow-up of heart failure patients at home according to the organizational model established by the national health insurance administration as part of the national PRADO program for heart failure patients. Its goal is to support heart failure patients returning home from the hospital and to optimize their management. The PRADO INCADO project integrates a telemedicine solution to further and better structure the patient’s care pathway, enabling healthcare professionals to exchange information with one another via the use of a telemonitoring solution.

In addition, the platform is capable of outlining the patient’s care pathway, which is a major theme in medicine for government and authorities. It likewise provides a means for various healthcare professionals to communicate with each other, while facilitating access to medical resources. With this in mind, an enhanced version of the E-care platform and MyPredi^TM^ AI is scheduled to be tested in CHF patients’ homes, as part of the so-called PRADO-INCADO project. PRADO is a French program to support patients returning home after hospitalization, with PRADO-INCADO specifically targeting the post-hospitalization period of HF patients (Figure 6) [32].

Over a period of several months, 200 patients with NYHA Class II to IV HF will be followed-up by means of the PRADO organizational model developed by the national health insurance administration for HF patients (to date, *n* = 35) [33].

For recruitment, the project will benefit from the HF care protocol that includes cardiologists, internists, emergency physicians and geriatricians from the Strasbourg University Hospital [37,43]. Here again, no draconian selection of patients will occur. Instead, patients will be gradually enrolled so that they are deemed representative of CHF subjects across France. Within this care protocol, the mean age of patients we are likely to enroll is 82 years [36]. Both morbidity and mortality data from the 100 patients using the telemedicine solution will be compared to those of patients enrolled into the PRADO program (*n* = 100), as well as to those of patients eligible for neither the PRADO-INCADO project nor the PRADO program, representing the control group (*n* = 100). The first results are expected to be available during the first quarter of 2019.

The patients will likely originate from the greater Strasbourg area. In addition to its medical objectives, the PRADO-INCADO project also includes features having to do with cost analyses and organizational aspects concerning patient care pathways and the implementation of the solution by both patients and healthcare professionals.

The data derived from the PRADO INCADO project are to be augmented with data from the patient’s environment along with the patient’s profile, including prior history, medication, and adherence to treatment, diet, and lifestyle guidelines, as well as the use of the system itself. The merging of this data will likely render the telemonitoring system more effective [41]. This phase should represent an in-depth study enabling us to improve diagnosis by aided machine learning and therefore to detect abnormalities at an earlier stage.

This is in keeping with the research of Mortazavi et al. on the utility of AI in managing HF patients, and particularly the possibility afforded by AI for predicting re-hospitalization for acute HF [39].

### 3.4. First Positive Randomized Study of Second-Generation Telemedicine: The TIM-HF2 Study

Koehler et al. reported the results of the large home telemonitoring study for HF in The Lancet, namely, The Telemedical Interventional Management in Heart Failure II (TIM-HF2) [34]. This is the first EBM study clearly showing that remote patient management over the course of twelve months reduced the number of days lost due to unplanned cardiovascular hospital admissions, with the differences between groups being statistically significant and clinically relevant. This outcome was predominantly driven by a reduction in cardiovascular deaths. Home telemonitoring triggered several potentially life-saving hospital admissions and overall slightly reduced the number of days patients were hospitalized due to HF.

Between 13 August 2013 and 12 May 2017, 1571 patients were randomly assigned to either remote patient management (*n* = 796) or standard care (*n* = 775) [34]. Of these patients, 765 in the remote patient management group and 773 in the standard care group actually began their assigned care, being included in the full analysis set. The mean patient age was 70 years, and most were men. As a baseline, all patients exhibited a left ventricular ejection fraction of <45% and NYHA Class II or III HF while receiving treatment with diuretics. About 60% of patients were living in rural areas of Germany.

**Figure 7:**
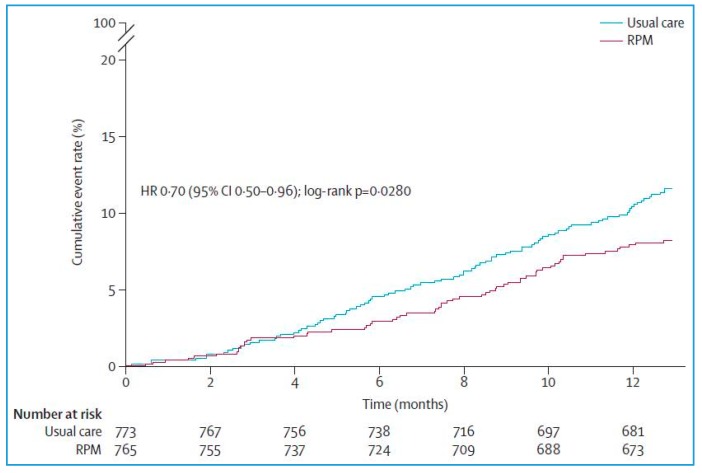
TIM-HF2 study (*n* = 1515). Rate of cumulative events in patients randomly assigned to remote patient management (*n* = 796) or usual care (*n* = 775); RMP = Remove Patient Management (adapted from [34]).

The percentage of days lost due to unplanned cardiovascular hospital admissions and all-cause deaths (primary end point) was 4.88% (95% CI 4.55–5.23) in the remote patient management group vs. 6.64% (6.19–7.13) in the standard care group (ratio 0.80, 95% CI: 0.65–1; *p* = 0.0460). Patients assigned to remote patient management lost a mean of 17.8 days (95% CI: 16.6–19.1) per year compared with 24.2 days (95% CI: 22.6–26) per year for patients assigned to standard care. The all-cause death rate was 7.86 (95% CI: 6.14–10.10) per 100 person years of follow-up in the remote patient management group vs. 11.34 (95% CI: 9.21–13.95) per 100 person years of follow-up in the standard care group (Hazard Ratio [HR] 0.70, 95% CI: 0.5–0.96; *p* = 0.0280) (Figure 7). Cardiovascular mortality did not significantly differ between groups (HR 0.671, 95% CI: 0.45–1.01; *p* = 0.056).

The study used a noninvasive, multi-parameter telemonitoring system installed in the patient’s home, consisting of a three-channel ECG, a blood pressure monitoring device and a weight scale, by means of which the information was transferred remotely [34]. Patients received a mobile phone in order to contact the telemedical center in case of emergency. Patients were likewise followed via monthly phone interviews.

For this TIM-HF2 care strategy, the key component was a well-structured telemedical center with physicians and HF nurses (“coordination center”) available 24 h a day and 7 days a week with the ability to act promptly according to the individual patient risk profile. The actions taken by the telemedical center staff included changes in medication and admissions to the hospital as needed, in addition to educational activities.

In our opinion, this telemedical center and the process put in place explained a large part the results observed in the TIM-HF2 study.

## 4. Perspectives Regarding New Developments in Telemedicine

### 4.1. New Evaluation Criterion of Telemedicine Projects in the CHF Field

To our knowledge, the E-care project with MyPredi^TM^ AI is the first to be conducted among a non-selected real-life CHF population including elderly patients (mean age: 72 years) with multiple concomitant comorbidities and diseases attested by a mean Charlson index of 4.1 over a long follow-up period of more than one year [22,33].

Moreover, this telemedicine solution has been demonstrated to allow situations likely to progress to acute HF to be detected at an early stage [43,44]. Here, we are entering the realm of predictive medicine, which should be further personalized for MyPredi^TM^ AI (i.e., better tailored to each patient’s phenotype). Of interest for practitioners is that the platform detected 100% of cardiac decompensations with alerts resulting in three-quarters of cases, and where only 10% of alerts did not directly relate to HF.

To our knowledge, this is the first connected, smart system to be developed with tools using new technologies, while prefiguring “telemedicine 2.0” solutions.

Considering the current problem of access to health professionals, this E-care platform is capable of structuring the patient’s care pathway, and is a major medical topic that should interest our government and authorities. Likewise, the E-care project provides a means for healthcare professionals to exchange information with each other, thereby facilitating patient access to medical resources.

In addition, the E-care project likely pursues other objectives such as the potential parameters of tomorrow’s telemedicine projects, as listed in Table 3.

Most of these objectives are considered to be either HF-related direct consequences or undesirable effects encountered by HF patients in a real-life setting.

### 4.2. Integration of the Problems of Elderly Subjects and Comorbidities Associated with HF

The challenge for “tomorrow’s” telemedicine is to develop new telemedicine projects and solutions, including the resolution of several medical problems and difficulties, such as [37]:

- The specificities (no appetite for new technologies and new uses) and problems (e.g., falls, malnutrition, mild cognitive impairment, etc.) of elderly subjects, who are the main subjects affected by chronic diseases;
- The co-existence of several chronic pathologies (e.g., CHF, DM, COPD, etc.) and comorbidities (e.g., arterial hypertension, renal failure, etc.) in the same individual, while providing comprehensive and “global” care for the individual in all medical and societal dimensions;
- The multiplicity of care structures and medical organizations (e.g., with or without human resources, telemedical centers, etc.);
- The significant logistical barriers to implementing tele-health (Many existing health systems are not designed for these technologies to be integrated within existing information systems).

In the chronic disease setting, new remote sensors and tailored questionnaires are presently being integrated into the telemedicine platform (e.g., E-care solutions for our team), including remote glucose meters, actimeters and electronic spirometers, along with new knowledge in the form of ontologies in order to enhance the telemedicine platform and broaden its utility to other chronic diseases like DM and COPD [44].

In this setting, additional personnel and specific protocols are necessary, which must be specific for each chronic disease and targeted for each patient, while allowing the possibility for each patient to exhibit more than one chronic disease. Most of these protocols must still be funded by means of existing resources or external grants.

These diseases share a number of commonalities with HF in terms of epidemiology and natural history. Along with HF, DM and COPD are among the most common diseases in developed countries, and thus represent a major public health concern for society [1,2]. Like HF, they are accompanied by frequent hospital admissions and re-admissions for well-known causes. These causal factors are detectable, enabling professionals to act ahead of time, as with CHF, thereby avoiding disease progression. Developing warning alerts for these chronic diseases should enhance the existing system.

Future research must also focus on the accessibility and practicality of telemedicine interventions.

Reimbursement also remains a major concern and a barrier (“glass ceiling”), given that much of the care delivered by telehealth is not covered by traditional fee-for-service payment models (e.g., in France, where all HF patients benefit from an integrated processing of healthcare expenses) [28,36]. The growth of value-based payment models may, however, provide incentives to implement telehealth as a strategy for providing high-quality, cost-effective and coordinated care [36].

At country levels, differences in medical practice laws, restrictions on how telehealth can be delivered, and patients that receive these services limit telemedicine’s applicability as well.

### 4.3. Telemedicine 2.0 in the Setting of DM

In addition to CHF, DM and metabolic disorders are other potential application fields for telemedicine that are being thoroughly investigated in France. Innovative projects are presently being developed, such as the PLASIDIA platform by the Centre Européen d’Etude du Diabète (European Center for the Study of Diabetes) in Strasbourg (France) [45].

To this end, we have developed an upgraded version of the E-care platform and MyPredi^TM^ AI (initially designed to follow CHF), which enables us to follow diabetic patients as part of the DIABETe project. The new version of the platform should be first deployed for “complex” diabetic patients, e.g., diabetic patients at high cardiovascular risk or diabetic patients treated with multiple injections [43]. Most of these patients have already developed or are likely to develop an HF episode, possibly followed by CHF over the long-term.

The DIABETe project seeks to detect the risk of hospitalization in diabetic patients at an early point in time, with patients classified as: (1) “very high cardiovascular risk” when presenting with a personal history of myocardial infarction (MI) or stroke, limb amputation or cardiomyopathy, and (2) receiving “intensive” insulin therapy, with at least three injections per day or pump administration, while offering them personalized follow-up and education regarding their illness and its management [43].

This population is particularly interesting, given that it allows for polypathology and polymedication to be studied, while requiring overall support. This population represents about 50% of diabetics hospitalized in diabetology and internal medicine departments (see above for the included population of patients in the E-care project). Apart from cardiovascular complications (myocardial infarction, arteritis obliterans of the lower limbs, etc.), these patients are frequently hospitalized for hypoglycemia, diabetes imbalance and infections as well as other complications.

The DIABETe project is based on an intelligent platform that likely assists healthcare professionals by automatically processing the information obtained from nonintrusive medical sensors (blood glucose meters, blood pressure monitors, actimeters, connected scales, etc.), as well as the subjective information provided by the patients themselves (via questionnaires) and his or her behavior (compliance), enabling early detection and reporting of situations at risk for hospitalization [43]. Patient and situation-adapted therapeutic education tools will be made available to the individual, and communication with the subject will likely occur via a touch pad.

Alerts indicating a deterioration of the patient’s condition will be generated by AI (new software version of MyPredi^TM^ adapted for the management of DM) and transmitted to the healthcare professionals in charge of the patient. The healthcare professional can thus anticipate decompensation and can initiate appropriate measures outside of an emergency setting.

These innovative solutions derived from new technologies should be readily accepted by patients. Medical data can likewise be shared among health professionals, being part of a city-wide hospital network. Ultimately, an improvement in the patients’ quality of life can be expected.

DIABETe does not compete with Diabeo^TM^ (version 1) or other expert systems aimed at optimizing the glycemic balance, which is per se the main objective of DM management [44,46]. The DIABETe project focuses on the “global” management of diabetes patients through the detection of situations carrying a risk of hospitalization: infection, cardiac decompensation, diabetic foot, as well as hypoglycemia and hyperglycemia episodes potentially leading to hospitalization.

Regarding the remote monitoring platform used in DIABETe, an integration of or interfacing with expert systems such as Diabeo^TM^ appears possible. As a reminder, the Diabeo^TM^ application, carried by Sanofi, was tested as part of the Télésage clinical study on 700 patients with Type 1 and Type 2 DM and treated with a basal-bolus regimen (multiple injections or pump) [46]. The primary endpoint of the Télésage study was HbA1c variation (glycemic control) at one year.

## 5. Points of Interest for the Clinician

To our opinion, this short narrative review supports, in a clinical pragmatic view, the efficacy of telemonitoring CHF patients, especially those with Classes III and IV NYHA and instable CHF. Several studies on CHF telemonitoring, using diverse technologies and transmitting different clinical, medical and behavioral data were found. Significant impacts were observed, mainly at the behavioral, clinical and structural levels. Minimal technical problems and cost-effectiveness analyses were reported.

- Close management of CHF patients through telemonitoring;
- Several improvements in control of HF;
- A better appropriation of the disease by the patients;
- A greater adherence to therapeutic and hygiene-dietary measures;
- A better patient quality of life;
- Good receptiveness by the patients and patient empowerment.

To date, the magnitude of its effects remains debatable, especially with the variation in patients’ characteristics (e.g., background, ability for self-management, medical condition), sample selection and approach for treatment of control groups.

In this setting, TIM-HF2 is the first positive prospective randomized study in regards to EBM with positive significant clinical benefit, in terms of unplanned cardiovascular hospital admissions and all-cause deaths.

To our opinion, the results of TIM-HF2 study may be mainly explained by telemedical center and the process put in place in this study. In fact, TIM-HF2 includes a well-structured telemedical coordination center with physicians and HF nurses, available 24 h a day and 7 days a week, with the ability to act promptly according to the individual patient risk profile. The actions taken by the telemedical center staff included changes in medication and admissions to the hospital as needed, in addition to educational activities.

## 6. Conclusions

Although numerous first-generation and second-generation non-invasive telemonitoring projects have been conducted in the CHF field, relatively few have been conducted within the “telemedicine 2.0” setting, using both ICT and Web 2.0 technologies. E-care telemonitoring is a project that entirely falls under this category, particularly when it includes the use of AI.

The projects’ potential utility in terms of morbidity, mortality and avoidance of hospital admissions is presently being extensively studied or documented. The projects’ impact in terms of health savings is likewise being assessed.

Indeed, although the earliest telemedicine projects have confirmed certain clinical benefits, they have mostly demonstrated its economic benefits. The TIM-HF2 study is the first to thoroughly document the interest of telemedicine in the CHF field, resulting in clinically relevant outcomes with statistical significance (percentage of days lost due to unplanned cardiovascular hospital admissions and all-cause deaths).

As with E-care or PRADO-INCADO, the “telemedicine 2.0” projects are perfectly compatible with the care pathways being developed for chronic diseases by the French health authorities, including the French ministry of health and the regional branch of the national health insurance administration.

Furthermore, all these findings should be analyzed with regard to the benefits of these telemedicine solutions, as outlined in Table 3.

This experience may enable us to personally witness the birth of “Tomorrow’s Medicine++”.

In the chronic disease field, given the epidemiology and expected shortage of time that careers provide, it appears essential to improve patient follow-up and education with better prevention, anticipation and, above all, improved selection of patients urgently needing the healthcare system.

## Figures and Tables

**Table 1:** Terms and definitions used in the field of telemedicine (adapted from [28]).

**Telemonitoring**	A telemedicine practice allowing a healthcare professional to remotely interpret the data necessary for the patient’s medical follow–up in order to make decisions regarding his or her care -remote data collection from a patient via a connected device or questionnaire to monitor his or her vital signs and symptoms at home on a daily basis.
**Teleexpertise**	The practice of telemedicine consisting, for a medical professional, of seeking the opinion of one or more medical professionals regarding elements of the patient’s medical file -the remote seeking, by a health professional, of a second medical opinion via the sending of images (scans, X-rays, eye fundus exams, etc.) and sometimes exchanges via Internet–based videoconferences
**Teleconsultation**	A telemedicine practice allowing a medical professional to have a remote consultation with a patient -in the context of a teleconsultation, a health professional assisting the remote professional, as well as a psychologist at the patient’s sidea second opinion consultation by a specialist.

**Table 2:** Signs and symptoms of heart failure.

Symptoms	Signs
Breathlessness Orthopnea Paroxysmal nocturnal dyspnea Reduced exercise tolerance Fatigue, tiredness, increased time to recover after exercise Ankle swelling Nocturnal cough Wheezing Confusion (especially in elderly patients) Palpitations Dizziness	Elevated jugular venous pressure Hepatojugular reflux Third heart sound (gallop rhythm) Laterally displaced apical impulse Weight gain (>2 kg/week) Peripheral edemas Tachycardia Tachypnea Hepatomegaly Oliguria

**Table 3:** Potential parameters to be evaluated in a telemedicine project focused on heart failure.

Overall Mortality	Therapeutic Education
Heart failure mortality	Hygiene-dietary and therapeutic compliance
Hospitalization for heart failure	Optimization of food and sports hygiene
Re-hospitalization for heart failure	Patient self-management
Number of hospitalization days	Optimization of the care pathway
Health costs	Structuring of the care pathway
Heart failure management costs	
Number of days off work	City-hospital relations
	Information sharing among health professionals
Quality of life	System use by health professionals
